# Monitoring and Retrofitting of Reinforced Concrete Beam Incorporating Refuse-Derived Fuel Fly Ash Through Piezoelectric Sensors

**DOI:** 10.3390/ma19020432

**Published:** 2026-01-22

**Authors:** Jitendra Kumar, Dayanand Sharma, Tushar Bansal, Se-Jin Choi

**Affiliations:** 1 Department of Civil Engineering, Sharda School of Engineering and Technology, Sharda University, Greater Noida 201310, India; 2022300731.jitendra@dr.sharda.ac.in (J.K.); tushar.bansal@sharda.ac.in (T.B.); 2 Department of Architectural Engineering, Wonkwang University, Iksan-si 54538, Jeollabuk-do, Republic of Korea

**Keywords:** damage, electromechanical impedance technique, reinforced concrete beam, retrofitting, structural health monitoring, SDG11

## Abstract

This paper presents an experimental framework that allows damage identification and retrofitting assessment in reinforced concrete (RC) beam with implemented piezoelectric lead zirconate titanate (PZT) sensors embedded into the concrete matrix. The study was conducted with concrete prepared from 30% refuse-derived fuel (RDF) fly ash and 70% cement as part of research on sustainable materials for structural health monitoring (SHM). Electromechanical impedance (EMI) was employed for detecting structural degradation, with progressive damage and evaluation of recovery effects made using root-mean-square deviation (RMSD) and conductance changes. Concrete beam specimens with dimensions of 700 mm × 150 mm × 150 mm and embedded with 10 mm × 10 mm × 0.2 mm PZT sensors were cast and later subjected to three damage stages: concrete chipping (Damage I), 50% steel bar cutting (Damage II), and 100% steel bar cutting (Damage III). Three retrofitting stages were adopted: reinforcement welding (Retrofitting I and II), and concrete patching (Retrofitting III). The results demonstrated that the embedded PZT sensors with EMI and RMSD analytics represent a powerful technique for early damage diagnosis, reserved retrofitting assessment, and proactive infrastructure maintenance. The combination of SHM systems and sustainable retrofitting strategies can be a promising path toward resilient and smart civil infrastructure.

## 1. Introduction

With the aging of infrastructure and increasing environmental loads, reinforced concrete (RC) structures need to be retrofitted to ensure performance, durability, and safety. As time passes, these structures will be affected by steel-reinforcement corrosion, environmental factors, physical degeneracy, cracking, and inappropriate designs or construction measures. The focus therefore has rapidly shifted toward sustainable and resilient structural maintenance, retrofitting, and continuous health monitoring of concrete structures.

Structural health monitoring (SHM) is a revolutionary method for enhancing safety and facilitating early interventions required to retain structures in service for extended periods. The use of smart materials, specifically, piezoelectric sensors, in SHM systems has enabled proactive maintenance, particularly in the context of retrofitting. Conventionally, the evaluation and maintenance of RC structures have largely depended on visual examination and manual inspection. However, the associated tests are subjective, limited in their coverage, and may overlook internal problems such as reinforcement corrosion. Corrosion remains one of the most relatively common causes of structural damage in RC structures, responsible for over 74% of structural damage cases [1]. Further, such damage is typically identified only after substantial material deterioration has occurred, which limits conventional assessment techniques. To overcome these drawbacks, the civil engineering community has investigated sensor-based SHM systems that can enable continuous real-time monitoring and data-driven maintenance planning.

Among advanced sensing technologies, piezoelectric sensors have received considerable attention for monitoring minute variations in the stress, strain, and vibration characteristics of structural members. These sensors take advantage of the piezoelectric effect, in which an electric charge is produced in response to the application of a mechanical stress, providing a reliable approach for embedded structural monitoring [2]. The main advantage is that they can operate under varying environmental conditions and can be integrated into structural members. Some recent studies have utilized the radiation of elastic waves to detect the presence of cracks, delamination, and early-stage corrosion in RC beams and columns using piezoelectric impedance sensing and Lamb wave propagation method [3]. The results have shown that piezoelectric sensors can be an effective alternative to visual monitoring and conventional nondestructive testing techniques because they provide calibrated and quantifiable information regarding the integrity of structures. The development of smart aggregates, particularly piezoelectric-based sensors that can be embedded into the concrete matrix during casting, can further increase the potential of SHM systems. These sensors can continuously record in situ stress–strain behaviors at the very beginning of damage initiation without requiring external energy or invasive protocols [1,4]. A previous study [5] reported a good linear relationship between the applied stress and electrical output in uniaxial piezoelectric sensors embedded in concrete, which is useful for accurate damage assessment and fatigue analysis. The emergence of sensor networks and wireless data communication systems has made SHM increasingly deployable and cost effective for large infrastructure systems.

Alongside the development of SHM, retrofitting of RC structures has attracted attention from both academia and industry. Most structures, particularly those that fail to comply with current earthquake-resistant codes, exhibit poor behavior following an earthquake and after prolonged periods under environmental loading. The purpose of retrofitting is to rehabilitate and/or improve the load-carrying capability, ductility, and durability of substandard structures. Among such exterior reinforcement methods, the combination of fiber-reinforced polymer (FRP) composites utilized for retrofitting has shown promise owing to their high strength-to-weight ratio, corrosion resistance, and ease of application. Awari [6] showed that RC beams strengthened using glass fiber-reinforced polymer sheets exhibited a 10–14% increase in the ultimate load-carrying capacity and a higher deflection capacity. Other innovative techniques that have helped substantially enhance the performance of RC members include high-performance fiber-reinforced concrete (HPFRC) jacketing and externally bonded reinforcement [7]. Sanhudo et al. [8] reported that HPFRC jacketing can help increase the ductility and energy dissipation of I-beam joints subjected to cyclic loading in seismic-resistant applications. Saad et al. [9] investigated the long-term effectiveness of a carbon fiber-reinforced polymer in preventing corrosion-induced distress and enhancing the strength of damaged RC columns and slabs.

Despite the successful structural enhancement achieved using these retrofitting methods, there remains one major bottleneck: the lack of in situ testing systems for validation during the rebound process and long-term bidirectional real-time monitoring after the retrofitting. This is where SHM becomes crucial. Piezoelectric sensors may be embedded into the retrofit material or structural base to monitor the response of retrofitted members to the service load, offering a glimpse of how the sensor alters their behavior. For example, Shendkar et al. [10] proposed a hybrid retrofitted structure system that adopts FRP sheets with embedded piezoelectric sensors to achieve strengthening functions while simultaneously performing the monitoring. This method not only ensures that the structure serves its target purpose but is also based on the identification of stress concentrations, debonding, or delamination, which may cause catastrophic failure. Furthermore, SHM systems based on piezoelectric sensors are being increasingly integrated with building information modeling (BIM) platforms to generate intelligent decision support for maintenance and rehabilitation. Awari [6] presented a concept in which machine-readable sensor data were directly integrated into BIM-based asset management systems. This makes it possible to schedule predictive maintenance based on the real-time state of a structure, thereby significantly reducing lifecycle costs and increasing the asset value of the structure.

From a sustainability standpoint, the smart monitoring of structural rehabilitation is closely aligned with global environmental goals. Prolonging the lifespan of existing infrastructure also means fewer new materials and therefore reduced carbon emissions and fewer resources employed [11]. In such scenarios, smart retrofitting can not only increase the structural strength but can also facilitate the formation of green infrastructure. In addition, the reliability of piezoelectric sensors in harsh environments makes them stable for long-term environmental use under conditions where corrosion is an issue (e.g., in marine or coastal structures) [12]. Along with intelligent monitoring, sustainable construction materials are being utilized to minimize the carbon output of infrastructure.

Refuse-derived fuel (RDF) ash, which is a potential supplementary cementitious material (SCM), is a residue from the incineration of municipal solid waste (MSW) in the context of waste-to-energy incineration, and represents a new SCM source for concrete [13]. For India, the increasing urbanization and population, currently estimated at 1.38 billion and expected to exceed 1.52 billion by 2036, means that solid waste collection is an immediate and growing concern [14,15]. Given that MSW generation is over 62 million tons per year, use of alternative waste, such as RDF, is necessary. RDF can be co-combusted with coal to enhance combustion efficiency [16,17] and used as an alternative fuel in cement kilns [18]. In addition, RDF fly ash has the potential to increase the early-age compressive strength of concrete [19], making it a good candidate for partial cement substitution. However, RDF fly ash chemically differs from typical SCMs containing heavy metals and has a greater variation in chemical properties. A deeper understanding of hydration processing is a prerequisite for its safe and efficient application in building construction. Few investigations have been conducted on the early-age hydration properties of concrete containing RDF fly ash. For long-term durability, it is important to investigate the hydration process of RDF fly ash with cementitious materials, particularly in the very early age hydration process [12]. The early stages of hydration can now be monitored owing to the recent progress in the SHM technique, particularly using the electromechanical impedance (EMI) method based on piezoelectric sensors. EMI provides immediate sensitivity to microstructural changes, allowing the determination of the setting time, strength development, and compatibility of materials within tight ranges [20]. Considering its successful application to plain concrete, the EMI technique has the potential for early hydration kinetics studies on RDF fly ash-based concrete [15]. This can help confirm the potential of RDF fly ash as an ecologically sustainable cement replacement and as a means of ensuring the integrity of structures [21].

Hence, this study has clear objectives for advancing smart-sensing technologies for RC structures. This study proposed an experimental framework that allows damage identification and retrofitting assessment in reinforced concrete (RC) beam with implemented piezoelectric lead zirconate titanate (PZT) sensors embedded into the concrete matrix.

## 2. Materials and Methods

### 2.1. Collection and Preparation of RDF Fly Ash

The RDF fly ash employed in this study was procured from an industrial brick manufacturing unit located in Uttar Pradesh, India; however, the specific site details are not disclosed in compliance with the confidentiality obligations of the associated company. RDF is produced by processing MSW to extract noncombustible materials and contaminants, producing a high-calorific-value fuel. This pretreatment leads to higher efficiencies and cleaner gas emissions compared with raw MSW burning [22]. The treated RDF is burned in a furnace at temperatures in the range of approximately 850–1100 °C, yielding two forms of ash: bottom ash (accreted to the base of the furnace) and fly ash (carried by flue gases).

For this study, bottom ash, called RDF fly ash, was received, dried, and sieved to filter out coarse particles before being used in concrete as an SCM. The present research work was conducted at the Department of Civil Engineering, Sharda University, Greater Noida, India.

### 2.2. Concrete Mix Preparation and Its Properties

Two types of concrete mixes were studied to determine the potential of RDF fly ash as a partial replacement for OPC. The first mix series was prepared using a control mix without any additives and with 100% OPC. The second blend incorporated 30% of RDF fly ash as a replacement for OPC, which has been previously shown to result in higher early-age performance [15]. The specific gravity and water absorption of the loose fine aggregate in both types of mixes were 2.64 and 1–2%, respectively, with a bulk density of 1730 kg/m^3^. Natural coarse aggregates with a maximum nominal size of 20 mm and specific gravity of 2.64 were also employed in the two mixes. The w/c ratio of both the mixes was maintained at 0.43. A superplasticizer was added at 0.3 wt% to improve workability. An elemental analysis of the RDF fly ash was also performed to provide information on the nature and components of the oxides and trace elements that could affect the hydration kinetics and strength of the concrete. These results are summarized in Table 1; clearly, oxygen and silicon contents are high, with higher atomic percentages indicating the presence of silicate phases (e.g., quartz). The high carbon content was probably due to the incomplete combustion and the presence of carbonaceous materials. Furthermore, the presence of calcium and aluminum suggests the possible formation of aluminosilicate or chemical carbonate substances, indicating potential pozzolanic materials. PIMS and trace elements (Na, Mg, K, and Cl) were detected at low levels, suggesting their inclusion in the mineral complexes.

The correlation between the chemical and elemental compositions (as well as the physical features) between OPC and RDF fly ash indicates the differences that affect their suitability for cementitious applications. From the oxide composition of the two materials, it is evident that OPC is rich in CaO (63%) as a major cementitious constituent, whereas RDF fly ash has a lower CaO content (17.6%) and higher amounts of pozzolanic compounds, such as SiO_2_ (49.7%) and Al_2_O_3_ (10.5%), compared with SiO_2_ (22%) and Al_2_O_3_ (5%) in OPC. These constituents imply that the RDF fly ash can participate in secondary pozzolanic reactions, if any occur. RDF fly ash also contains high levels of Fe (O), MgO, and SO_3_, which can influence color, strength development, and long-term durability. In the modified mix, with 30% OPC (initially 482.55 kg/m^3^) replaced by the RDF fly ash (144.76 kg/m^3^), the cement content was reduced to 337.78 kg/m^3^, whereas the water-to-cement ratio, aggregate content, water quantity, and superplasticizer dosage were kept the same in both mixes to maintain consistency in terms of workability and strength performance.

The result of the RDF fly ash is reported only for comparison with that of OPC, as it is typically expressed as oxides and has low or negligible carbon and trace elements detected. Considerable amounts of carbon and oxygen were found in the RDF fly ash, which was represented as 8.95/13.97, indicating 8.95% of weight percent and 13.97% of atoms, respectively. This implies that the remainder (91.05%) was due to the presence of light atoms other than C and low energy. This difference is also owing to the low atomic weight of carbon; therefore, a small mass corresponds to many atoms. A similar trend was then extrapolated for other light elements, such as oxygen (53.64%/62.80%) and sodium (3.48%/2.84%), and this helped describe the chemical signature of RDF fly ash, which also included minor components such as chlorine, magnesium, potassium, and iron. They contribute to the formation of both cementitious and pozzolanic phases; however, some, such as Cl and alkalis, may be of concern given their effects on concrete durability.

Overall, this study demonstrated that RDF fly ash, although compositionally dissimilar to OPC, exhibits oxide and elemental features that make it suitable for partial replacement in cement systems, subject to full control of its variability and influence on long-term performance.

### 2.3. Damage and Retrofitting Process

**Figure 1:**
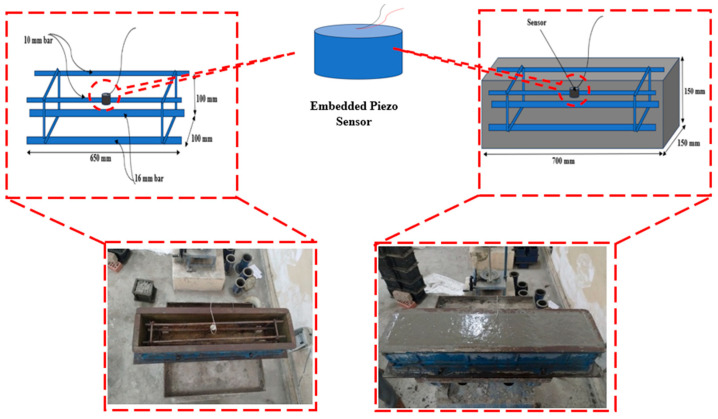
Casting and positioning of reinforced concrete.

An RC beam with dimensions of 700 mm (length) × 150 mm (width), and 150 mm (depth) was prepared to monitor the damage and retrofitting stages. The reinforcement included two high-yield-strength deformed bars with a diameter of 16 mm placed at the bottom, which served as the primary tension reinforcement, and two 10 mm bars at the top for compression. These reinforcements were assembled into cages with a vertical spacing of 100 mm and an effective bar cage length of 650 mm. An embedded piezoelectric sensor was attached at the top of the reinforcement bar to monitor the damage and retrofitting stages, as shown in Figure 1.

**Figure 2:**
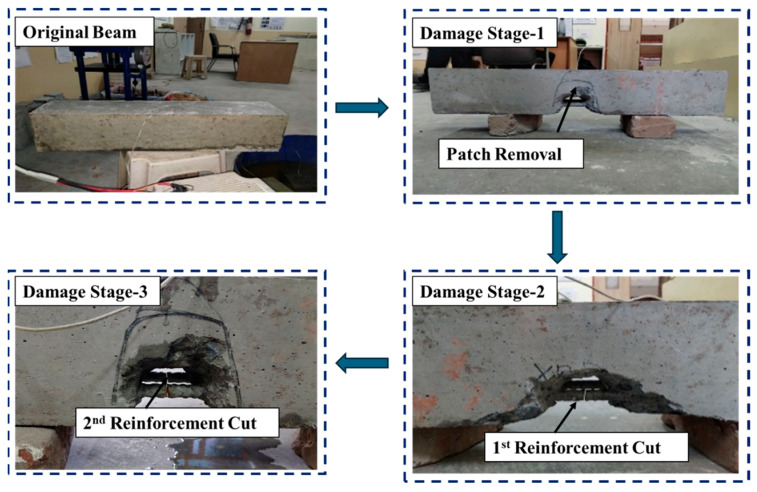
Damage stages in the reinforced concrete specimen.

A structured methodology was followed to simulate real-world degradation and monitor the response of RC beams to progressive damage and subsequent repair. The experimental procedure began with the casting of specimens and baseline data acquisition. Initial measurements were performed in the pristine stage, serving as a reference for all subsequent stages. Subsequently, a sequence of damages was introduced in three defined stages. In Damage Stage I, a concrete patch with dimensions of 150 mm × 150 mm × 75 mm was introduced at the mid-span, 300 mm from one end, to simulate damage. This action introduced a stress concentration and reduced the effective area resisting the load. In Damage Stage II, 50% of the bottom reinforcement (one 16 mm bar) was cut, significantly reducing the tensile resistance of the beam. In Damage Stage III, the remaining 16 mm bar was also cut, resulting in a 100% loss of bottom reinforcement and a complete compromise of flexural capacity. Figure 2 shows a pictorial representation of all the damage stages.

**Figure 3:**
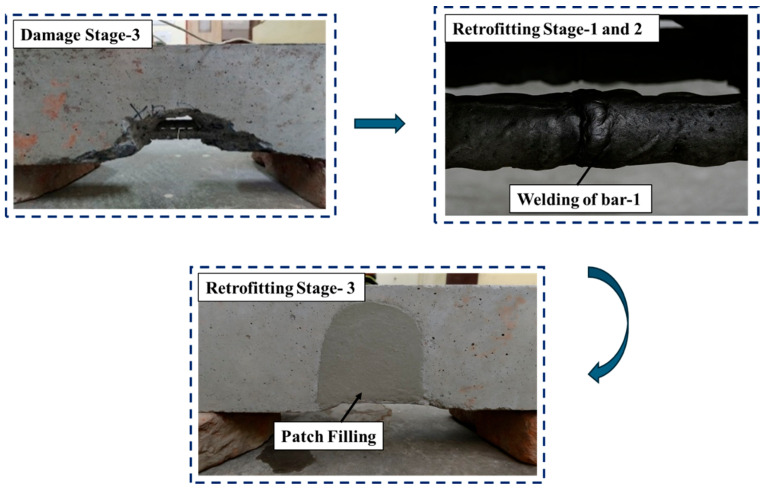
Retrofitting stages in the reinforced concrete specimen.

After simulating the damage, the beams underwent a three-stage retrofitting process. In retrofitting stages I and II, both the bottom reinforcement bars were welded back together to restore the load paths. Welding was performed at the exact points at which the bars were cut. In Retrofitting Stage II, the previously chipped notch was filled with freshly mixed concrete to restore the cross section of the beam and enhance its stiffness. These repair operations were monitored using the same embedded piezoelectric sensor, allowing for a comparative analysis of the structural response across the pristine, damaged, and retrofitted stages. Throughout each stage, EMI data were acquired to detect and quantify structural changes. Figure 3 shows a pictorial representation of all the retrofitting stages.

### 2.4. Data Acquisition Setup

**Figure 4:**
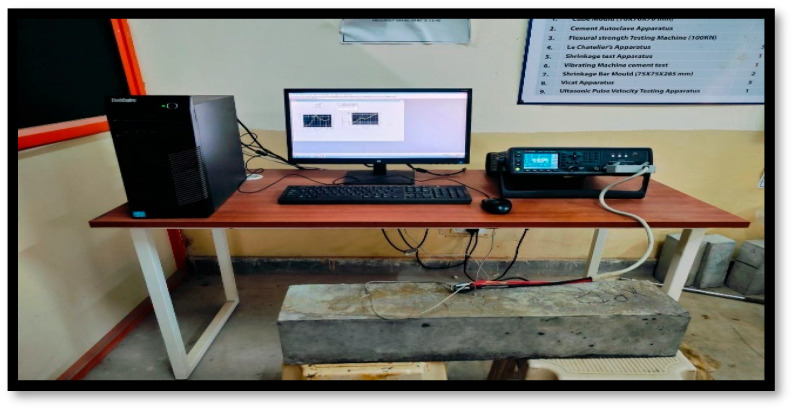
Data acquisition setup for reinforced concrete specimen.

Data in the form of EMI signatures were acquired using an inductance–capacitance-resistance meter, which was connected to the embedded piezoelectric sensor attached inside the RC beam, as shown in Figure 4. The data acquisition stages were the pristine stage, damage stage I, damage stage II, damage stage III, retrofitting stage I, retrofitting stage II, and retrofitting stage III. Any shift or deviation in the EMI signature from the baseline was interpreted as a sign of structural deterioration, or conversely, improvement in the case of successful retrofitting.

### 2.5. Statistical Analysis

To quantify the changes in EMI signatures, statistical parameters, such as the root mean deviation (RMSD), which has been established in numerous SHM fields, were calculated. The RMSD can be expressed by Equation (1):

(1) $$RMSD\left(\%\right)=\sqrt{\frac{\sum_{i=1}^{N}{{(G}_{i}-G_{bl})}^{2}}{{\sum_{i=1}^{N}\dot{{(G}_{bl})}}^{2}}}\times 100$$

Here, *G* is the conductance or resistance of the PZT patch during the hydration process ($G_{i}$) and baseline value ($G_{bl}$), and *i* represent the frequency index.

## 3. Results and Discussion

### 3.1. Analysis Based on EMI Signatures Using Piezoelectric Sensor

**Figure 5:**
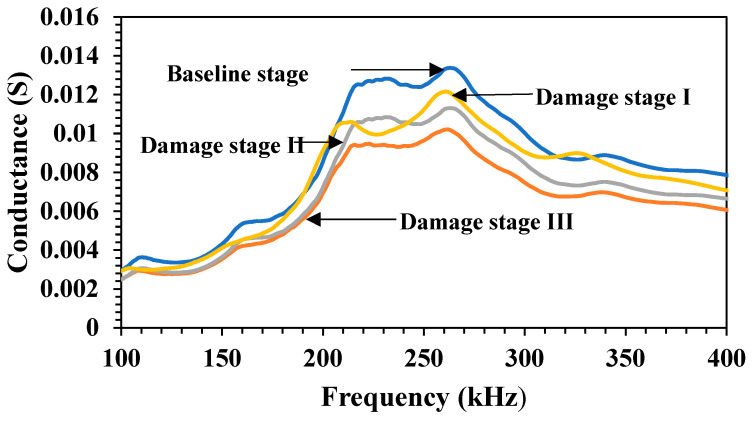
Conductance variation with frequency for a specimen during damage stages.

Figure 5 shows the variation in the conductance across the frequency range of 100–400 kHz for the RC beam in different damage stages. Clearly, the conductance signatures during the damage stages shifted in the downward direction compared with the baseline signature. At the baseline stage, the peak conductance was approximately 0.0135 S, which occurred at 265 kHz, indicating a healthy structure. As the damage initiated (Damage Stage I), the peak conductance decreased slightly to 0.012 S, and the peak shifted slightly leftward to 258 kHz, indicating a minor loss in stiffness. With further damage in Stage II, the conductance peak dropped to 0.010 S, and the peak frequency shifted more prominently reflecting moderate stiffness loss and increased damping. In Damage Stage III, which represented severe damage, the peak conductance decreased further to 0.009 S, and the frequency peak shifted to 264 kHz. This progressive leftward and rightward shift in frequency (from 265 to 258 kHz) and the decline in the peak conductance (from 0.0135 to 0.009 S) clearly indicated a loss of structural integrity. The greater the structural damage, the lower and flatter the conductance curve became, confirming the sensitivity of the EMI method in detecting even subtle changes in structural health.

**Figure 6:**
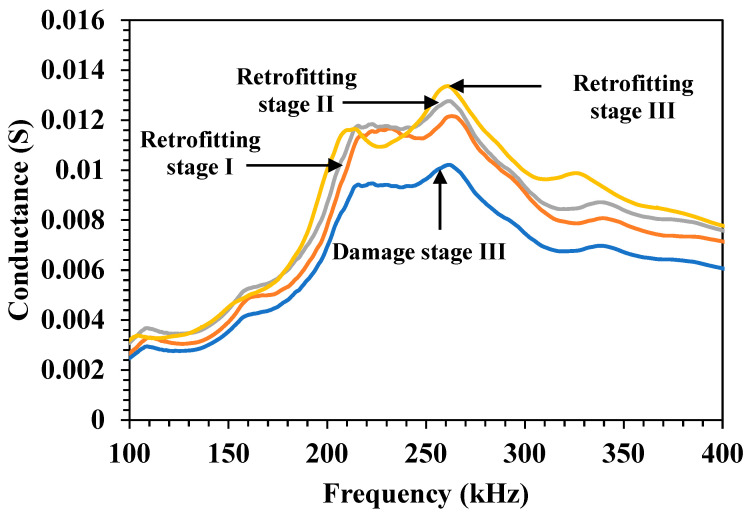
Conductance variation with frequency for the RC specimen during retrofitting process.

Figure 6 shows the variation in the conductance across the frequency range of 100–400 kHz for the RC beam during different retrofitting stages. Clearly, in Damage Stage III, representing the most damaged stage, the conductance significantly reduced, peaking at approximately 0.009 S in the frequency range of 240–265 kHz and then tapering off. This reduction in the peak amplitude compared with the other curves indicated severe structural degradation. Following this damage, the specimen showed partial recovery in Retrofitting Stage I, with the conductance increasing to approximately 0.0112 S near 269 kHz but still being lower than that in the subsequent stages. Further recovery was seen in Retrofitting Stage II, with the conductance peaking at 0.0126 S at 264 kHz, indicating better stiffness restoration and improved material coupling. Finally, the highest conductance was seen in Retrofitting Stage III across all frequencies, reaching a peak of 0.0131 S near 264 kHz and remaining consistently higher in the 200–300 kHz range.

This progressive increase in the conductance values at each retrofitting stage clearly illustrated the recovery of the structure and improved integrity, validating the efficiency of retrofitting in restoring damaged materials. The conductance shifts and increased peak values correlated directly with the enhanced electromechanical coupling and structural stiffness restoration.

### 3.2. Analysis Results of Statistical Parameters

**Figure 7:**
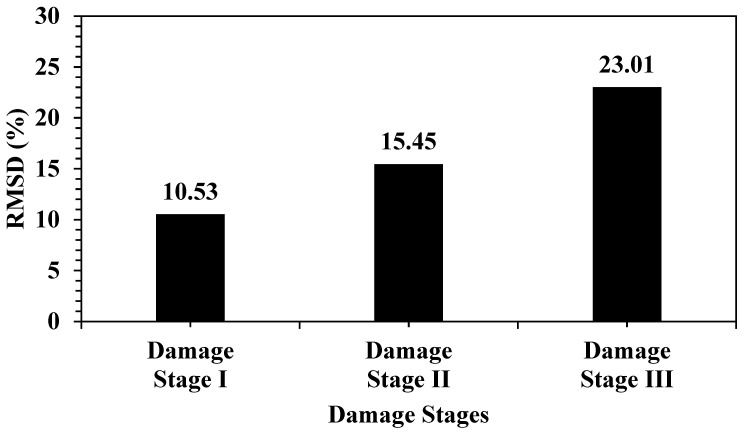
RMSD index during the damage process.

Figure 7 shows the evolution of the structural damage across the three defined stages. In Damage Stage I, the RMSD value was 10.53%, which indicated a moderate deviation from the baseline, corresponding to the early signs of damage. In Damage Stage II, the RMSD increased to 15.45%, reflecting further deterioration and a greater deviation in the impedance response. In Damage Stage III, the RMSD peaked at 23.01%, indicating significant structural degradation and the highest level of damage among the three stages. This progressive increase clearly demonstrated the sensitivity of the system to incremental damage, with a nearly 12.5% increase in the RMSD from Stages I to III.

**Figure 8:**
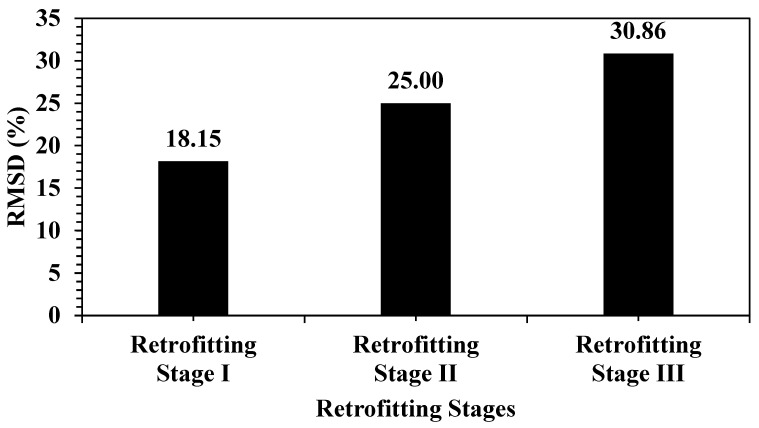
RMSD index during retrofitting process.

Figure 8 shows the evolution of the EMI response of the structure during retrofitting, indicating a recovery trend relative to the damaged stage. In Retrofitting Stage I, the RMSD increased to 18.15%, which, although still higher than that in the damaged stage, suggests a shift in structural properties owing to initial repair actions. In Retrofitting Stage II, the RMSD reached 25.00%, reflecting further deviation owing to additional retrofitting materials or techniques. In Retrofitting Stage III, the RMSD value peaked at 30.86%, which indicated the highest deviation from the original baseline, likely owing to substantial structural reinforcement or modification. This trend confirms that EMI sensing is highly responsive not only to damage but also to retrofitting, making it effective in monitoring both deterioration and repair activities.

## 4. Conclusions

This study demonstrated the use of piezoelectric PZT sensors for the real-time damage monitoring and retrofitting evaluation of RC elements using RDF fly ash as a sustainable cement replacement. Quantifiable changes in the structural behavior were identified by employing the EMI method, along with induced damage and staged RC beam retrofitting. A conductance analysis showed a striking shift in the data, reducing from above 0.0135 S (healthy) to less than 0.009 S (fully damaged), accompanied by a leftward/rightward shift in the frequency from 270 to 250 kHz, indicative of a worsening case of softness. The electromechanical responses were further validated inductance–capacitance using the RMSD index. The RMSD increased from 10.53% in Damage Stage I to 23.01% in Damage Stage III, confirming the sensitivity of the sensor to incremental damage. The effectiveness of the retrofitting was evident from the gradual restoration of the conductance signatures and further shifts in the RMSD. Specifically, the conductance values improved to 0.0145 S during Retrofitting Stage III, surpassing even the original baseline. The RMSD values during retrofitting increased from 18.15% (Stage I) to 30.86% (Stage III), not as a sign of new damage but as a reflection of stiffness enhancement due to material addition and repair interventions.

Overall, the integration of smart sensors and sustainable cementitious materials proved to be effective damage detection and quantitative assessment of retrofitting. These findings support the development of intelligent, resilient infrastructure systems and promote the use of eco-friendly materials, such as RDF fly ash, in modern civil engineering practices.

## Figures and Tables

**Table 1:** Integrated data on chemical properties, mix design, and elemental composition of RDF fly ash [13,15,16,22].

Parameter/Element Name	Unit/Description	OPC	RDF Fly Ash	Remarks
Loss on ignition (LOI)	%	1.69	1.72	Indicator of unburnt material or volatile content
CaO	%	63	17.6	Major cementitious component
Silicon dioxide (SiO_2_)	%	22	49.7	Major pozzolanic oxide
Al_2_O_3_	%	5	10.5	Enhances reactivity
Fe_2_O_3_	%	3.30	5.68	Minor oxide affecting color and strength
MgO	%	2.12	4.55	Can impact soundness of concrete
SO_3_	%	1.42	3.27	High sulfate may affect durability
K_2_O	%	0.71	2.73	Alkali component
TiO_2_	%	0.46	1.29	Present in trace amounts
P_2_O_5_	%	0.001	0.932	Trace element in RDF fly ash
ZnO	%	0.003	0.170	Trace element in RDF fly ash
Water-to-cement ratio	—	0.43	0.43	Constant for both mixes
Cement	kg/m^3^	482.55	337.78	Reduced by 30% in RDF mix
RDF fly ash	kg/m^3^	—	144.76	30% replacement of OPC
Fine aggregates	kg/m^3^	932.33	932.33	Constant
Coarse aggregates	kg/m^3^	744.38	744.38	Constant
Water	kg/m^3^	207.5	207.5	Constant
Superplasticizer	% by weight of cement	0.3	0.3	Constant
C	Weight %/Atomic %	—	8.95/13.97	Due to organic or incomplete combustion
O	Weight %/Atomic %	—	53.64/62.80	Indicates high oxide content
Na	Weight %/Atomic %	—	3.48/2.84	Trace mineral phase
Mg	Weight %/Atomic %	—	1.41/1.08	Trace compound
Al	Weight %/Atomic %	—	7.97/5.53	Supports aluminosilicate formation
Si	Weight %/Atomic %	—	13.18/8.79	Indicates silicate dominance
Cl	Weight %/Atomic %	—	0.51/0.27	Possibly from waste combustion
K	Weight %/Atomic %	—	2.08/1.00	Minor alkali metal
Ca	Weight %/Atomic %	—	5.88/2.75	Cementitious contribution
Fe	Weight %/Atomic %	—	2.90/0.97	Minor oxide in ash

## Data Availability

The original contributions presented in this study are included in the article. Further inquiries can be directed to the corresponding authors.

## Abbreviations

The following abbreviations are used in this manuscript:

SHM	Structural health monitoring
RDF	Refuse-derived fuel
BIM	Building information modeling
SCM	Supplementary cementitious material
